# A Novel Approach to Manage Hypercholesterolemia: Veterans Affairs Lipid Optimization Reimagined Quality Improvement Program

**DOI:** 10.1016/j.jacadv.2026.102935

**Published:** 2026-06-18

**Authors:** Luc Djousse, Rachel E. Ward, Helen Marucci-Wellman, Nedim Yel, Kristin Colson, Abigail A. Santos, Mason Coleman-Lopez, Eddie Pan, Tharen Leesch, David Peña, Michelle Congdon, Michele Bolles, David Gagnon, J. Michael Gaziano

**Affiliations:** a MAVERIC, Boston VA Healthcare System, Boston, MA, USA; b Brigham and Women’s Hospital and Harvard Medical School, Boston, MA, USA; c American Heart Association, Dallas, TX, USA; d Boston University School of Public Health, Boston, MA, USA

**Keywords:** atherosclerosis, cardiovascular disease, dyslipidemia, epidemiology, quality improvement, statins

## Abstract

**Background:**

Elevated serum low-density lipoprotein cholesterol (LDL-C) is a major risk factor for atherosclerotic cardiovascular disease (ASCVD). Despite the availability of effective drugs to lower LDL-C, only one-third of veterans with ASCVD have LDL-C treated to goal.

**Objectives:**

This study aimed to determine if a variety of lipid optimization strategies including a health coach tailored to unique barriers and needs can improve: 1) the percentage of veterans with LDL-C <70 mg/dL; and 2) the usage and patients’ adherence to any lipid-lowering therapy (LLT).

**Methods:**

Veterans Affairs Lipid Optimization Reimagined Quality Improvement was embedded in the usual clinical care of 167,505 veterans with prevalent ASCVD inclusive of 88,778 veterans with baseline LDL-C ≥70 mg/dL in 50 Veterans Affairs Medical Centers between December 2022 and September 2025. At each Veterans Affairs site, American Heart Association consultants worked with local providers to: 1) identify barriers to lipid management; and 2) implement strategies tailored to the identified barriers. The primary outcome was the proportion of veterans with LDL-C <70 mg/dL between baseline and follow-up LDL-C measurement at 24 months.

**Results:**

A total of 88,778 veterans were included in current analyses with mean age of 70.0 (SD: 10.5) years and 6,250 (7.0%) women. Overall, 32.9% of veterans achieved LDL-C <70 mg/dL and for 24,610 veterans with 24-month LDL-C data, 34.4% achieved LDL-C goal (all *P* < 0.001). The mean LDL-C decreased by 16.6 mg/dL; use of LLT increased from 77.4% to 87.8%; and adherence to any LLT increased from 56.3% to 65.1% between baseline and 24 months.

**Conclusions:**

This program showed that addressing site-specific barriers to lipid management is associated with improved LDL-C among Veterans with ASCVD.

Elevated low-density lipoprotein cholesterol (LDL-C) is a major and treatable risk factor for atherosclerotic cardiovascular disease (ASCVD)^1^^,^^2^ in the general population and among US veterans.^3^ ASCVD is one of the leading causes of morbidity and mortality^2^ among U.S. veterans despite the availability of effective drugs to treat dyslipidemia (eg, statins) and access to a wide range of network providers across the nation. ASCVD is also associated with reduced quality of life and high health care costs.^2^ Previous studies have suggested that veterans have a disproportionately higher burden of ASCVD than nonveterans.^4^, ^5^, ^6^, ^7^ Adults with prevalent ASCVD are at higher risk of recurrent events,^8^, ^9^, ^10^ thereby underscoring the importance of secondary prevention. Similar to nonveterans, about two-thirds of veterans with ASCVD have serum LDL-C above the recommended target^3^ and remain at higher risk of recurrent events given the log-linear relation between serum LDL-C and ASCVD.^11^ Contributing factors to suboptimal LDL-C among veterans with ASCVD may include poor adherence to lipid-lowering therapy (LLT), drug intolerance, suboptimal dosage of LLTs, limited education on the impact of LDL-C on ASCVD outcomes, and perhaps inconsistencies across existing lipid guidelines, among others. Of note is that while current practice guidelines for dyslipidemia management from the Veterans Affairs (VA)/Department of Defense (DoD) recommend moderate-dose statins for primary ASCVD prevention and high-dose statins with intensification by increasing statin dose, adding ezetimibe, and then adding additional agents such as protein convertase subtilisin/kexin type 9 inhibitors, inclisiran, or bempedoic acid in high-risk patients; VA/DoD guidelines do not recommend target cholesterol levels to guide those decisions.^12^ However, according to the 2018 American Heart Association (AHA)/American College of Cardiology (ACC) Guidelines on the Management of Blood Cholesterol, it is reasonable to add ezetimibe therapy to very high-risk patients with clinical ASCVD who are on maximally tolerated statin therapy and have LDL-C level of 70 mg/dL or higher (Class of Recommendation; IIa and Level of Evidence: B-R).^13^ Furthermore, routine lipid monitoring is not recommended by the VA/DoD guidelines contrary to the AHA/ACC guidelines which recommend LDL-C measurement to help assess adherence and efficacy of treatment.^14^

The existing high prevalence of veterans with ASCVD and dyslipidemia and resulting high burden and costs to the VA underscore the urgent need for innovative and effective strategies to reduce the burden of dyslipidemia and its costly downstream consequences. To this end, the Veterans Affairs Lipid Optimization Reimagined Quality Improvement (VALOR-QI) Program (a joint collaboration between VA and AHA) was designed to address this critical gap in lipid management among high-risk veterans with ASCVD with the primary objective to increase the proportion of veterans with ASCVD and LDL-C <70 mg/dL during the program, given the reported benefits of statins on both primary and secondary prevention.^15^ By identifying site-specific barriers that prevent achievement of target LDL-C, designing and implementing strategies to address barriers, and identifying effective solutions to the problem, VALOR-QI has the potential to greatly reduce the burden of and costs related to dyslipidemia within the VA system and improve the cardiovascular health of millions veterans in a cost-effective and sustainable manner.

## Methods

### Design

VALOR-QI is a new collaborative program between the VA and the AHA focused on lipid optimization among U.S. veterans with ASCVD and dyslipidemia (i.e., fasting or nonfasting LDL-C ≥70 mg/dL) upon engagement. VALOR-QI is an uncontrolled preimplementation-postimplementation evaluation embedded in routine clinical care within the VA. A detailed description of design and methods of the VALOR-QI program has been previously published.^16^

### Participating veterans and sites

We used the Corporate Data Warehouse^17^ to identify adult veterans with prevalent ASCVD (myocardial infarction, stroke, peripheral arterial disease, history of coronary artery bypass, percutaneous coronary intervention, carotid artery surgery, and peripheral artery surgery using International Classification of Diseases-9 and -10 codes as previously reported)^18^, ^19^, ^20^, ^21^, ^22^ and planned upcoming appointment with a provider within one of the eligible VA sites as described in the design paper.^16^ We used available LDL-C (fasting and nonfasting) data closest to the patients’ first visit to the program from electronic health records to identify veterans with baseline serum LDL-C ≥70 mg/dL and subsequent LDL-C values measured during the program for current analyses. Veterans were engaged in the program through 5 phases. The term engaged refers to a time window when a veteran is seen by a provider who: 1) is participating in VALOR-QI; and 2) has begun receiving advice/education materials from the AHA consultants/coordinating center.

### Intervention

**Figure 1:**
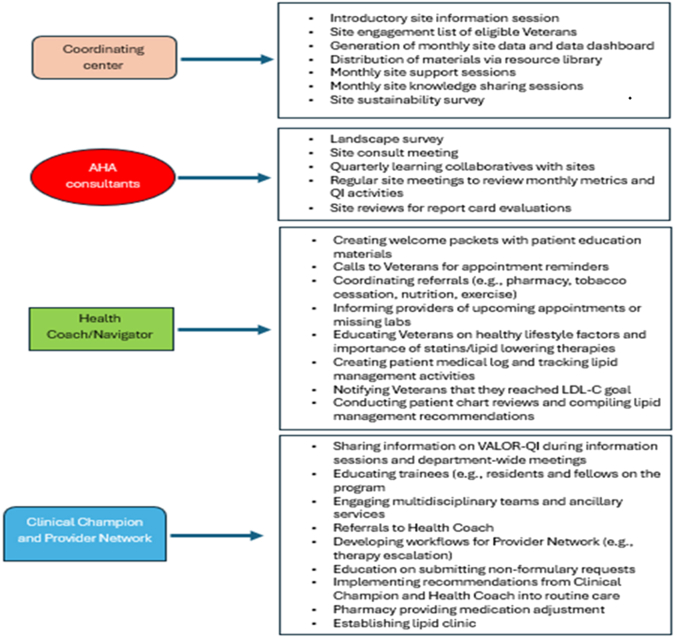
**Summary of Interventions Implemented in Veterans Affairs Lipid Optimization Reimagined Quality Improvement** AHA = American Heart Association; LDL-C = low-density lipoprotein cholesterol; VALOR-QI = Veterans Affairs Lipid Optimization Reimagined Quality Improvement.

A detailed description of the methods used to identify site-specific barriers and strategies to address those barriers has been previously published.^16^ An important feature of VALOR-QI was the use of health coaches/navigators in multiple roles to assist both veterans (ie, calls to remind veterans of upcoming clinic and lab appointments, notifying veterans when they achieved LDL-C goal, or veterans’ education on the importance of healthy lifestyle factors and importance of LLT) and health providers achieve lipid optimization. Health coaches/navigators used in the program were required to have college graduate-level education, although some were nurses. Sites were provided monthly site data reports using electronic health record data. These reports included aggregate data on demographics, LDL-C levels, and LLT use of engaged veterans at their site. Sites were also given access to a central electronic VALOR-QI Network Resource Library that contained various educational and engagement materials and learning modules. An overview of VALOR-QI intervention strategies is summarized in Figure 1.

### Outcomes

While this program supported maintenance of LDL-C levels among those who had already achieved LDL-C below 70 mg/dL, the primary outcome of this program was the proportion of veterans who reached LDL-C below 70 mg/dL among those not at target at baseline after 2 years of the implementation of QI activities. This was computed as the proportion of veterans with LDL-C <70 mg/dL at the end of 24 months among those with baseline LDL-C ≥70 mg/dL; the denominator for assessing LDL-C <70 mg/dL and absolute change in LDL-C was restricted to veterans with both baseline and 24-month LDL-C. The secondary outcomes included absolute change in LDL-C computed as LDL-C at 24 months minus LDL-C at engagement; relative change in LDL-C computed as the difference in LDL-C between 24 months and engagement divided by LDL-C at engagement; prevalence of any LLT use and adherence to any LLT assessed using the proportion of days covered (PDC). PDC was calculated as the sum of days covered for all LLT prescriptions released to veterans during the 24-month follow-up, divided by the number of days in the follow-up period. LLT adherence was defined as a PDC ≥0.80.

### Statistical analyses

Continuous variables were expressed as mean (standard deviation) if normally distributed or as median (interquartile range) if non-normally distributed. Categorical variables are presented as percentages. *P* values comparing pre- and postintervention outcomes were computed using paired sample *t*-test for continuous variables and McNemar’s test for categorical variables. To check if variance estimates were affected by within/across station variability and also to control for confounding, we repeated main analyses using a multilevel approach. Specifically, statistical analysis system *proc glimmix* procedure was used for generalized mixed linear models with LDL-C values at the 2 different time points modeled as the outcome and VA site number as a random variable. We controlled for sex, age, race, and calendar time of the start of the phase in the program in the model. In secondary analyses, we also repeated main analyses stratified by sex, baseline LDL-C (≥100 and ≥160 mg/dL), and restricted to veterans aged 75+ years at engagement. We also contrasted baseline characteristics of veterans with and without 24-month LDL-C to evaluate potential bias due to missing LDL-C at 2 years. All analyses were completed using R version 4.4.1. This manuscript followed Standards for QUality Improvement Reporting Excellence publication guidelines (Central Illustration).^23^

**Central Illustration:**
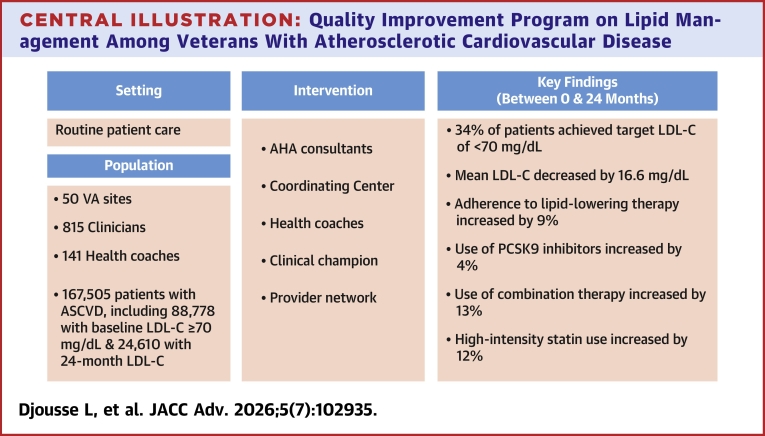
**Quality Improvement Program on Lipid Management Among Veterans With Atherosclerotic Cardiovascular Disease** This quality improvement program was associated with lipid optimization among veterans with atherosclerotic cardiovascular disease. ASCVD = atherosclerotic cardiovascular disease; AHA = American Heart Association; LDL-C = low-density lipoprotein cholesterol; VA = Veterans Affairs; PCSK9 = protein convertase subtilisin/kexin type 9.

## Results

### Participants’ characteristics

Between December 2022 and September 2025, a total of 167,505 veterans with prevalent ASCVD were engaged in VALOR-QI. Current analyses are based on a subset of 88,778 veterans with baseline LDL-C ≥70 mg/dL inclusive of 24,610 veterans with 24-month LDL-C measurement (Supplemental Figure 1). The 88,778 veterans were engaged in 5 phases: 19,733 were in Vanguard (phase 1); 14,060 in phase 2; 17,007 in phase 3; 20,141 in phase 4; and 17,837 in phase 5. The mean age was 70.0 (SD: 10.5) years and mean body mass index was 30.0 (SD: 6.2); the majority were men (n = 82,528 [93.0%]); with respect to race, 61,101 (68.8%) were White and 19,396 (21.8%) were Black; the prevalence of smoking at baseline was 22,896 (25.8)% (Table 1). Major barriers identified included staffing shortages, poor adherence to any LLT, clinical inertia, and lack of patient and professional education on lipid management and lifestyle modification.

**Table 1:** Baseline Characteristics of the 88,778 VALOR-QI Participants With Baseline LDL-C≥ 70 mg/dL by Recruitment Phase

	Total Population (N = 88,778) [1/22 to 8/23]^a^	Vanguard (n = 19,733) [1/22 to 12/22]	Phase 2 Sites (n = 14,060) [7/22 to 3/23]	Phase 3 Sites (n = 17,007) [12/22 to 5/23]	Phase 4 Sites (n = 20,141) [2/23 to 7/23]	Phase 5 Sites (n = 17,837) [2/23 to 8/23]
Age (y)	69.9 (10.5)	69.4 (10.5)	69.1 (10.6)	70.00 (10.3)	70.3 (10.3)	70.5 (10.6)
Age 75+ y at baseline (%)	33,626 (37.9%)	7,122 (36.1%)	4,855 (34.5%)	6,450 (37.9%)	7,861 (39.0%)	7,338 (41.1%)
Sex (% women)	6,250 (7.0%)	1,407 (7.1%)	1,154 (8.2%)	1,372 (8.1%)	1,327 (6.6%)	990 (5.6%)
Race (%)						
White	61,101 (68.8%)	13,925 (70.6%)	8,009 (57.0%)	11,784 (69.3%)	14,033 (69.7%)	13,350 (74.8%)
Black	19,396 (21.8%)	3,796 (19.2%)	4,454 (31.7%)	3,682 (21.7%)	4,235 (21.0%)	3,229 (18.1%)
Others	8,281 (9.3%)	2012 (10.2%)	1,597 (11.4%)	1,541 (9.1%)	1873 (9.3%)	1,258 (7.1%)
BMI (kg/m^2^)	30.0 (6.2)	30.2 (6.3)	29.8 (6.2)	30.1 (6.2)	30.0 (6.1)	30.0 (6.1)
Current smoking (%)	22,896 (25.8%)	5,367 (27.2%)	3,729 (26.5%)	4,611 (27.1%)	4,504 (22.4%)	4,685 (26.3%)
Veterans Integrated Service Network (VISN)	1, 2, 4, 5, 6, 7, 8, 9, 10, 12, 15, 17, 21, 22, 23	1, 2, 8, 10, 12, 15, 17, 21, 22	7, 9, 19, 21, 22	1, 5, 6, 7, 9, 12, 16, 22	1, 5, 7, 8, 10, 16, 19, 21, 22	1, 2, 4, 5, 8, 10, 12, 15, 16, 23
Health coaches (n)	141	37	33	28	27	16
Health care providers (n)	815	129	140	168	180	198
LDL-C (mg/dL)	104.9 (31.7)	104.1 (31.0)	106.2 (31.9)	106.2 (33.5)	104.9 (31.7)	103.5 (30.6)
Triglycerides (mg/dL)	141.9 (91.1)	142.9 (93.3)	137.0 (88.2)	148.3 (95.7)	140.3 (89.30)	140.8 (88.5)
Total cholesterol (mg/dL)	176.1 (38.5)	175.2 (38.1)	177.9 (39.3)	178.0 (39.4)	175.4 (38.7)	175.0 (37.1)
Systolic BP (mm Hg)	132.1 (18.8)	131.5 (18.9)	131.9 (18.8)	132.1 (18.6)	132.4 (19.1)	132.3 (18.6)
Diastolic BP (mm Hg)	75.8 (10.3)	76.3 (10.3)	76.1 (10.5)	75.6 (10.5)	75.2 (10.4)	76.0 (10.0)
Statin use (%)	56,837 (64.0%)	12,874 (65.2%)	8,857 (63.0%)	10,898 (64.1%)	13,284 (66.0%)	10,924 (61.2%)
Ezetimibe use (%)	7,275 (8.2%)	1,572 (8.0%)	946 (6.7%)	1,614 (9.5%)	1739 (8.6%)	1,404 (7.9%)
Fibrates use (%)	1759 (2.0%)	419 (2.1%)	325 (2.3%)	313 (1.8%)	344 (1.7%)	358 (2.0%)
PCSK9 use (%)	1,326 (1.5%)	231 (1.2%)	153 (1.1%)	363 (2.1%)	335 (1.7%)	244 (1.4%)
Use of other LLT (%)^b^	807 (0.9%)	198 (1.0%)	101 (0.7%)	211 (1.2%)	157 (0.8%)	140 (0.8%)

Values are mean (SD) or n (%).

BMI = body mass index; BP = blood pressure; LDL-C = low-density lipoprotein cholesterol; LLT = lipid-lowering therapy; PCSK9 = protein convertase subtilisin/kexin type 9; VALOR-QI = Veterans Affairs Lipid Optimization Reimagined Quality Improvement.

a Dates (time when the first and the last champions in each phase were identified and accepted their roles in the program).

b Other LLT include fish oil, inclisiran, etc.

### Primary outcome

Overall, 32.9% of veterans included in current analyses achieved serum LDL-C <70 mg/dL by the end of the program, irrespective of the follow up (*P* < 0.001). The corresponding proportion was 34.4% among 24,610 veterans who participated in the program long enough to have 24-month LDL-C measurement (Table 2). Using multilevel model with adjustment for potential confounding factors did not alter the results (ie, in unadjusted analyses, mean LDL-C changed from 104.2 mg/dL at baseline to 87.6 mg/dL at 24 months [a change of −16.55 mg/dL]; corresponding values using adjusted multilevel models were 108.5 mg/dL at baseline and 92.0 mg/dL at 24 months [a change of −16.60 mg/dL) (Supplemental Table 1).

**Table 2:** Change in LDL-C, LLT Use, and Adherence to Any LLT Use Over 24 Months in 24,610 Veterans in VALOR-QI

	Baseline (n = 24,610)	At 24 Mo (n = 24,610)	*P* Value
Mean LDL-C (SD), mg/dL	104.2 (31.2)	87.6 (36.1)	<0.001
Prevalence of LDL-C <70 mg/dL	0 (0%)	8,461 (34.4%)	<0.001
Absolute change of LDL-C from baseline (mg/dL)	0	−16.6 (37.4)	<0.001
Prevalence of LLT use	19,044 (77.4%)	21,617 (87.8%)	<0.001
Adherence to any LLT use (PDC)^a^	6,533 (56.3%)	7,551 (65.1%)	<0.001

PDC = proportion of days covered; other abbreviations as in Table 1.

a PDC: proportion of days covered, calculated as the sum of days covered for all LLT prescriptions released to Veterans during the 24-month follow-up, divided by the number of days in the follow-up period; LLT adherence was defined as a PDC ≥0.80.

### Secondary outcomes

The mean serum LDL-C was 104.2 (SD, 31.2) mg/dL at baseline and 87.6 (SD, 36.1) mg/dL at 24 months (absolute decrease of 16.6 mg/dL and corresponding relative decrease of 16.0%, *P* < 0.001) (Table 2). The prevalence of any LLT use was 77.4% at baseline and 87.8% at 24 months (Table 2) and such association was noticeable for the use of statin in general, high-intensity statin, ezetimibe, protein convertase subtilisin/kexin type 9 inhibitors inclusive of inclisiran, and combination therapy (Supplemental Figure 2). Adherence to any LLT was 56.3% at baseline and 65.1% at 24 months (*P* < 0.001).

### Stratified analyses

When baseline and 24-month data were analyzed among 8,266 veterans aged 75+ years at baseline, 37.1% of them achieved LDL-C <70.0 mg/dL (*P* < 0.001); mean LDL-C was 99.0 (SD, 27.6) mg/dL at baseline and 83.4 (SD, 32.7) mg/dL at 24 months, *P* < 0.001; and adherence to any LLT was 61.9% at baseline and 68.5% at 24 months (*P* < 0.001), Table 3. Change in LDL-C between baseline and 24 months was larger among veterans with higher baseline LDL-C (for example, we observed a −30.5 mg/dL and −63.2 mg/dL change in mean LDL-C in veterans with baseline LDL-C ≥100 mg/dL and ≥160 mg/dL, respectively, Supplemental Figure 3). A smaller proportion of women (21.1%) than men (35.5%) achieved LDL-C<70 mg/dL at 24 months. In addition, there was a lower usage of any LLT and adherence to any LLT in women (Supplemental Table 2). Lastly, baseline characteristics of veterans with and without LDL-C measurement at 24 months were comparable (Supplemental Table 3).

**Table 3:** Change in LDL-C, LLT Use, and Adherence to Any LLT Over Time in 8,266 LOR-QI Subjects Aged 75+ at Baseline

Outcomes	Baseline	At 24 Mo	*P* Value
Mean LDL-C (mg/dL)	99.0 (27.6)	83.4 (32.7)	<0.001
Prevalence of LDL-C <70 mg/dL	0 (0%)	3,070 (37.1%)	<0.001
Absolute change of LDL-C from baseline (mg/dL)	0	−15.6 (34.0)	<0.001
Prevalence of LLT use	6,336 (76.7%)	7,110 (86.0%)	<0.001
Adherence to any LLT use (PDC)^a^	2,427 (61.9%)	2,682 (68.5%)	<0.001

PDC = proportion of days covered; other abbreviations as in Table 1.

a PDC: proportion of days covered, calculated as the sum of days covered for all LLT prescriptions released to Veterans during the 24-month follow-up, divided by the number of days in the follow-up period; LLT adherence was defined as a PDC ≥0.80.

## Discussion

Our data from this quality improvement program show that the use of multipronged approaches to address barriers to lipid optimization was associated with about 34.4% achievement of LDL-C <70 mg/dL, 16.6 mg/dL drop in serum mean LDL-C, and about 10% higher prevalence of LLT use and adherence to any LLT among U.S. veterans with ASCVD and dyslipidemia. As expected, the drop in serum LDL-C was larger in veterans with higher baseline LDL-C. Finally, we demonstrated that the program had similar associations among men, women, and veterans aged 75+ years at baseline.

VALOR-QI is the first and largest quality improvement program implemented across the VA network to improve lipid optimization among nearly 170,000 high-risk veterans. Although the program will continue for the assessment of long-term benefits, it is important to emphasize that the proportion of veterans with ASCVD achieving LDL-C below 70 mg/dL after 2 years (34%) is larger than corresponding values observed in secular trends analyses in 1.2 million veterans with ASCVD between 2002 (15%) and 2020 (40%) or about 1.25% per year (a difference of 25% improvement in 19 years); furthermore, while secular trends in statin use among veterans with ASCVD have been steady around 70% between 2002 and 2020, VALOR-QI program showed 88% statin use among veterans with ASCVD (personal communication, unpublished data except abstract [AHA 2023; https://doi.org/10.1161/circ.147.suppl_1.P4]). The large LDL-C change observed in this program could have a great clinical significance given the log-linear relation between LDL-C and ASCVD,^11^ one of the leading causes of death and disability among veterans.^2^^,^^22^ These important questions will be addressed in the future after additional years of follow-up. It is noteworthy that the higher proportion of: 1) veterans with optimal LDL-C; 2) LLT usage; and 3) adherence to any LLT and a lower mean LDL-C at 24 months were also observed in veterans aged 75+ years; a subgroup of the population in whom lipid guidelines remain equivocal partly due to limited data on older adults who have been less likely to be included in previous lipid trials.^24^ Nonetheless, these findings are in line with our previous reports demonstrating that statin use was associated with a lower risk of death and ASCVD among older veterans^21^^,^^25^^,^^26^ and older U.S. male physicians.^27^ If confirmed in the ongoing large trial of statin therapy in older adults,^28^ our findings provide support in favor of a more aggressive lipid management even among 75+ year-old veterans. Strategies implemented in VALOR-QI included the use of a health coach/navigator as a team member with multiple functions within the local team of network providers. A total of 141 health coaches/navigators were hired and utilized in VALOR-QI program and we believe that those coaches/navigators contributed significantly to the success of our program. Additional elements of the intervention included veterans’ education on healthy lifestyle factors (not smoking, physical activity, or healthy diet) and importance of LLT; flagging high-risk patients for upcoming clinic visits using the existing electronic health record system; creation of lipid clinics; educating trainees (ie, fellows and residents), developing workflows for networks (ie, LLT escalation), providing monthly data reports to sites using electronic health record data, etc. All these elements listed above and in Figure 1 are simple tools that are already part of the health care process or can easily be incorporated into the workflow at the point of care for ASCVD patients, thereby assuring the sustainability and uptake of the interventions by other medical centers within and outside the VA network.

### Study Limitations

The current project has some limitations. First, unlike a clinical trial where subjects are randomized to treatment or placebo and a causal inference can be made, VALOR-QI utilized an observational pre/postdesign built in routine clinical care and cannot establish causality. Second, VALOR-QI was not designed to assess the impact of the program on hard ASCVD endpoints. Thus, we are unable to directly appraise the impact of our program on the actual number of ASCVD events prevented. However, such data will be obtained in the future expansion of the program and continuing follow-up leveraging electronic health records. Third, as a quality improvement program, participating veterans were not required to provide blood samples at frequent intervals for LDL-C measurements. Hence, there is a possibility that our LDL-C data were biased if ordering lipid panels was influenced by ASCVD risk, comorbidity, and specialty of the health care provider. The fact that we observed: 1) comparable baseline characteristics of veterans with and without 24-month LDL-C (Supplemental Table 3; 2) lower mean LDL-C and higher usage and adherence to any LLT across a wide range of baseline LDL-C is reassuring that our findings may not be completely explained by such bias. Fourth, it is possible that a small number of the veterans participating in VALOR-QI had their lipids measured outside the VA system or moved out of the VALOR-QI network. These scenarios could have led to incomplete data on LDL-C and secondary outcomes at 24 months and underestimation of the magnitude of the association of our program with outcomes. Fifth, we have not yet evaluated the impact of the program on health care costs. However, we will be reporting on cost analyses in future reports. Sixth, the current publication unit did not focus on the impact of VALOR-QI on LDL-C among veterans with missing baseline LDL-C (n = 8,211) or those with baseline LDL-C <70 mg/dL. While subsequent publications will address those questions, there is no doubt that VALOR-QI impacted veterans without baseline LDL-C in terms of bringing them to the clinic for LDL-C control. This conjecture is supported by the fact that 1,291 out of 8,211 veterans with ASCVD and without LDL-C at baseline (15.7%) had at least 1 LDL-C measurement and 546 (6.7%) had 2 or more LDL-C measurements during the program. Lastly, our program was implemented prior to the new ACC/AHA lipid guidelines^29^ with target LDL-C of <55 mg/dL. While our program targeted LDL-C < 70 mg/dL, 15.3% of the 24,610 with 24-month LDL-C were treated to <55 mg/dL.

## Conclusions

Findings from VALOR-QI showed that a program with a health coach/navigator addressing site-specific barriers to lipid optimization is associated with a high proportion of veterans with ASCVD and dyslipidemia achieving LDL-C <70 mg/dL. The fact that tools and strategies used in VALOR-QI are simple, relatively inexpensive, and accessible to providers and patients at each point of care in the VA system assures sustainability of the program and a high likelihood of improved veterans’ cardiovascular health. Future work could expand this program to the entire VA and non-VA health care systems to maximize potential benefits and assess the impact of the program on hard ASCVD outcomes. This type of program utilizing a health coach/navigator, providing regular data reports from the electronic health record, conducting learning collaboratives, and providing educational materials and resources (eg, on the importance of LLTs and lifestyle modifications) could be applied to other preventive interventions/management such as hypertension, type 2 diabetes, etc.

Perspectives **COMPETENCY IN PRACTICE-BASED LEARNING:** A multipronged quality improvement program based on identification and addressing site-specific barriers to lipid management can favorably impact lipid management among veterans with atherosclerotic cardiovascular disease and dyslipidemia. The intervention was associated with a lower serum low-density lipoprotein cholesterol, higher proportion of (i) veterans with low-density lipoprotein cholesterol <70 mg/dL, (ii) usage of high-intensity statin and combination therapy, and (iii) adherence to any lipid-lowering therapy. These findings emphasize that a low-cost approach which uses a health coach to engage patients in the management of their lipids could be effective and sustainable in reducing the burden of dyslipidemia in at-risk patients.

**TRANSLATIONAL OUTLOOK 1:** Our findings suggest that targeting high-risk patients with dyslipidemia with a health coach and addressing barriers facing clinicians can be impactful in improving cardiovascular health.

**TRANSLATIONAL OUTLOOK 2:** Future studies should include a comparator to further evaluate the efficacy of such intervention on hard endpoints and long-term sustainability of the program.

## Funding support and author disclosures

The VALOR-QI program is supported by Novartis Pharmaceuticals Corporation. Support for VA/Centers for Medicare & Medicaid Services data is provided by the Department of Veterans Affairs, VA Health Services Research and Development Service, VA Information Resource Center (project numbers SDR 02-237 and 98-004). This article does not represent the views of the Department of Veterans Affairs or the U.S. government. The funders/sponsors had no role in the design and conduct of the program; collection, management, analysis, and interpretation of the data; preparation, review, or approval of the manuscript; and decision to submit the manuscript for publication. Dr Djousse has received grants from the U.S. Highbush Blueberry Council. All other authors have reported that they have no relationships relevant to the contents of this paper to disclose.

## References

[1] Dawber T.R., Kannel W.B., Revotskie N., Stokes J., Kagan A., Gordon T. (1959). Some factors associated with the development of coronary heart disease: six years' follow-up experience in the Framingham study. Am J Public Health Nations Health.

[2] Martin S.S., Aday A.W., Allen N.B. (2025). 2025 Heart Disease and Stroke Statistics: a report of US and global data from the American Heart Association. Circulation.

[3] Nguyen X.T., Quaden R.M., Wolfrum S. (2018). Prevalence of ideal cardiovascular health metrics in the Million Veteran Program. Am J Cardiol.

[4] Assari S. (2014). Veterans and risk of heart disease in the United States: a cohort with 20 years of follow up. Int J Prev Med.

[5] Hinojosa R. (2019). Veterans' likelihood of reporting cardiovascular disease. J Am Board Fam Med.

[6] Vance M.C., Wiitala W.L., Sussman J.B., Pfeiffer P., Hayward R.A. (2019). Increased cardiovascular disease risk in veterans with mental illness. Circ Cardiovasc Qual Outcomes.

[7] Ebrahimi R., Yano E.M., Alvarez C.A. (2023). Trends in cardiovascular disease mortality in US women veterans vs civilians. JAMA Netw Open.

[8] Chen G., Farris M.S., Cowling T. (2021). Prevalence of atherosclerotic cardiovascular disease and subsequent major adverse cardiovascular events in Alberta, Canada: a real-world evidence study. Clin Cardiol.

[9] Hurford R., Wolters F.J., Li L. (2020). Prevalence, predictors, and prognosis of symptomatic intracranial stenosis in patients with transient ischaemic attack or minor stroke: a population-based cohort study. Lancet Neurol.

[10] Wannamethee S.G., Shaper A.G., Lennon L. (2004). Cardiovascular disease incidence and mortality in older men with diabetes and in men with coronary heart disease. Heart.

[11] Robinson J.G., Jayanna M.B., Bairey Merz C.N., Stone N.J. (2020). Clinical implications of the log linear association between LDL-C lowering and cardiovascular risk reduction: greatest benefits when LDL-C >100 mg/dl. PLoS One.

[12] O'Malley P.G., Arnold M.J., Kelley C. (2020). Management of dyslipidemia for cardiovascular disease risk reduction: synopsis of the 2020 updated U.S. Department of Veterans Affairs and U.S. Department of Defense Clinical Practice Guideline. Ann Intern Med.

[13] Rao S.V., O'Donoghue M.L., Ruel M. (2025). 2025 ACC/AHA/ACEP/NAEMSP/SCAI Guideline for the management of patients with acute coronary syndromes: a report of the American college of Cardiology/American Heart Association Joint Committee on clinical practice Guidelines. Circulation.

[14] Al Rifai M., Blumenthal R.S., Stone N.J. (2021). Department of Veterans Affairs (VA) and U.S. Department of Defense (DoD) guidelines for management of dyslipidemia and cardiovascular disease risk reduction: putting evidence in context. Prog Cardiovasc Dis.

[15] Guo B.Q., Li H.B., Zhao B., Xu P.W. (2025). Comparative efficacy of LDL-C-Lowering therapies in first-time versus recurrent myocardial infarction prevention: a meta-analysis of large-scale randomized controlled trials. Eur J Prev Cardiol.

[16] Santos AAD J., Fink A., Seth J. (2025). The Veterans Affairs lipid optimization reimagined - quality Improvement Program: rationale and methods. J Biomed Res Environ Sci.

[17] Fihn S.D., Francis J., Clancy C. (2014). Insights from advanced analytics at the Veterans Health Administration. Health Aff (Millwood).

[18] Imran T.F., Kurgansky K.E., Patel Y.R. (2019). Serial sodium values and adverse outcomes in heart failure with preserved ejection fraction. Int J Cardiol.

[19] Song R.J., Larson M.G., Aparicio H.J. (2023). Moderate alcohol consumption on the risk of stroke in the Million Veteran Program. BMC Public Health.

[20] Honerlaw J.P., Ho Y.L., Nguyen X.T. (2020). Fried food consumption and risk of coronary artery disease: The Million Veteran Program. Clin Nutr.

[21] Orkaby A.R., Lu B., Ho Y.L. (2024). New statin use, mortality, and first cardiovascular events in older US Veterans by frailty status. J Am Geriatr Soc.

[22] Lu B., Posner D., Vassy J.L. (2022). Prediction of cardiovascular and all-cause mortality after myocardial infarction in US veterans. Am J Cardiol.

[23] Ogrinc G., Davies L., Goodman D., Batalden P., Davidoff F., Stevens D. (2016). SQUIRE 2.0 (Standards for QUality Improvement Reporting Excellence): revised publication guidelines from a detailed consensus process. BMJ Qual Saf.

[24] Cholesterol Treatment Trialists C. (2019). Efficacy and safety of statin therapy in older people: a meta-analysis of individual participant data from 28 randomised controlled trials. Lancet.

[25] Orkaby A.R., Driver J.A., Ho Y.L. (2020). Association of Statin use with all-cause and cardiovascular mortality in US veterans 75 years and older. JAMA.

[26] Barayev O., Hawley C.E., Wellman H. (2023). Statins, mortality, and major adverse cardiovascular events among US veterans with chronic kidney disease. JAMA Netw Open.

[27] Orkaby A.R., Gaziano J.M., Djousse L., Driver J.A. (2017). Statins for primary prevention of cardiovascular events and mortality in older men. J Am Geriatr Soc.

[28] Joseph J., Pajewski N.M., Dolor R.J. (2023). Pragmatic evaluation of events and benefits of lipid lowering in older adults (PREVENTABLE): trial design and rationale. J Am Geriatr Soc.

[29] Writing Committee M., Blumenthal R.S., Morris P.B. (2026). 2026 ACC/AHA/AACVPR/ABC/ACPM/ADA/AGS/APhA/ASPC/NLA/PCNA Guideline on the management of dyslipidemia: a report of the American college of Cardiology/American Heart Association Joint Committee on Clinical Practice Guidelines. Circulation.

