# Supplementary material for: A Novel Approach to Manage Hypercholesterolemia: Veterans Affairs Lipid Optimization Reimagined Quality Improvement Program
Source: JACC Adv. 2026 Jun 18;5(7):102935. doi: 10.1016/j.jacadv.2026.102935 (PMC13311920; doi:10.1016/j.jacadv.2026.102935)
Supplement: Supplemental_Material [file mmc1.pdf]

Supplemental Table 1. Multilevel mixed model estimates of change in LDL-C, LLT use, and adherence to LLT use between baseline and 24 months in VALOR-QI (n = 24610) with 95% Confidence Intervals

**Outcomes**	**Adjusted* baseline mean/% (95%CI)'**	**Adjusted* Closest to 24 month mean/% (95% CI)**	**P**
Mean LDL-C (SD), mg/dL	108.5 (107.1 ,109.9)	92.0 (90.5 , 93.4)	<0.001
Prevalence of LDL-C <70 mg/dL	0 (0%)	28.0% (25.9 ,30.2)	<0.001
Absolute change of LDL-C from baseline (mg/dL)		-16.6 (-17 , -16)	<0.001
Prevalence of LLT use	74.4% (72.7 ,76.0)	84.4% (82.6 ,86.3)	<0.001
Adherence to LLT use (PDC)[a]	51.2% (48.9 ,53.5)	59.1% (56.5 ,61.8)	<0.001

*Multilevel Model controlling for Station, Gender, Race, Age and calendar quarter of first visit in program

Supplemental Table 2. Change in LDL-C, LLT use, and adherence to LLT use between baseline and 24 months in VALOR-QI 24610 subjects stratified by sex

	**Men (n=22725)**				**Women (n=1885)**		
**Outcomes**	**Baseline**	**At 24 months**	**p**		**Baseline**	**At 24 months**	**p**
Mean LDL-C (mg/dL)	103.22 (30.61)	86.34 (35.41)	<0.001		115.31 (36.04)	102.71 (40.54)	<0.001
Prevalence of LDL-C <70 mg/dL	0 (0%)	8063 (35.5%)	<0.001		0 (0%)	398 (21.1%)	<0.001
Absolute change of LDL-C from baseline (mg/dL)	0	-16.88 (37.06)	<0.001		0	-12.60 (40.54)	<0.001
Prevalence of LLT use	17800 (78.3%)	20130 (88.6%)	<0.001		1244 (66.0%)	1487 (78.9%)	<0.001
Adherence to LLT use (PDC)*	6174 (56.7%)	7132 (65.5%)	<0.001		359 (50.6%)	419 (59.1%)	<0.001

*PDC: Proportion of days covered, calculated as the sum of days covered for all LLT prescriptions released to Veterans during the 24-month follow-up, divided by the number of days in the follow-up period; LLT adherence was defined as a PDC ≥ 0.80.

Supplemental Table 3. Baseline characteristics of veterans with and without 24-month LDL-C

**Characteristics**	**No 24-month LDL-C (N=64,168)**	**Available 24-month LDL-C (N=24,610)**	**Total population (N=88,778)**
Mean age (SD), y	70.15 (10.60)	69.18 (10.06)	69.88 (10.47)
Median age [Min, Max]	72.00 [23.00, 104.00]	71.00 [22.00, 103.00]	72.00 [22.00, 104.00]
Age Group			
18-54 (age at index)	5243 (8.2%)	2118 (8.6%)	7361 (8.3%)
55-64 (age at index)	12305 (19.2%)	5114 (20.8%)	17419 (19.6%)
65-74 (age at index)	21260 (33.1%)	9112 (37.0%)	30372 (34.2%)
75 and Above (age at index)	25360 (39.5%)	8266 (33.6%)	33626 (37.9%)
Race			
Black or African American	13858 (21.6%)	5538 (22.5%)	19396 (21.8%)
White	44432 (69.2%)	16669 (67.7%)	61101 (68.8%)
Other or unknown	5878 (9.2%)	2403 (9.8%)	8281 (9.3%)
Sex			
Male	59803 (93.2%)	22725 (92.3%)	82528 (93.0%)
Female	4365 (6.8%)	1885 (7.7%)	6250 (7.0%)

Ethnicity			
Hispanic or Latino	4399 (6.9%)	1479 (6.0%)	5878 (6.6%)
Not Hispanic or Latino	56408 (87.9%)	21836 (88.7%)	78244 (88.1%)
Unknown or Null	3361 (5.2%)	1295 (5.3%)	4656 (5.2%)
Rurality			
MultURH	126 (0.2%)	62 (0.3%)	188 (0.2%)
Rural	20837 (32.5%)	7108 (28.9%)	27945 (31.5%)
Urban	43169 (67.3%)	17421 (70.8%)	60590 (68.2%)
Missing	36 (0.1%)	19 (0.1%)	55 (0.1%)
Lipid Levels			
LDL-C Mean (SD)			
Baseline	105.15 (31.90)	104.15 (31.22)	104.87 (31.72)
Lipid Lowering Therapies			
Any			
12 months before/incl. index	40674 (63.4%)	19044 (77.4%)	59718 (67.3%)
Statin			
12 months before/incl. index	38709 (60.3%)	18128 (73.7%)	56837 (64.0%)
Ezetimibe			
12 months before/incl. index	4915 (7.7%)	2360 (9.6%)	7275 (8.2%)
PCKS9			
12 months before/incl. index	824 (1.3%)	502 (2.0%)	1326 (1.5%)

Resin			
12 months before/incl. index	196 (0.3%)	85 (0.3%)	281 (0.3%)
Fibrates			
12 months before/incl. index	1186 (1.8%)	573 (2.3%)	1759 (2.0%)
Other Chol			
12 months before/incl. index	510 (0.8%)	297 (1.2%)	807 (0.9%)
High-dose Statin			
12 months before/incl. index	25476 (39.7%)	11959 (48.6%)	37435 (42.2%)
Statin Comb.			
12 months before/incl. index	4792 (7.5%)	2395 (9.7%)	7187 (8.1%)
BMI, Total Cholesterol, and Triglycerides			
BMI			
Mean (SD)	29.87 (6.19)	30.40 (6.20)	30.02 (6.20)
Median [Min, Max]	29.15 [15.00, 59.97]	29.67 [15.02, 59.90]	29.30 [15.00, 59.97]
Missing	7664 (11.9%)	1700 (6.9%)	9364 (10.5%)
Total Cholesterol			
Mean (SD)	176.31 (38.69)	175.61 (37.96)	176.12 (38.49)
Median [Min, Max]	168.00 [50.00, 497.00]	168.00 [51.00, 475.00]	168.00 [50.00, 497.00]
Missing	5743 (9.0%)	2028 (8.2%)	7771 (8.8%)

Triglycerides			
Mean (SD)	141.47 (90.51)	143.03 (92.72)	141.91 (91.14)
Median [Min, Max]	117.00 [12.00, 1000.00]	118.00 [16.00, 981.00]	118.00 [12.00, 1000.00]
Missing	6659 (10.4%)	2291 (9.3%)	8950 (10.1%)
smoker			
CURRENT	16743 (26.1%)	6153 (25.0%)	22896 (25.8%)
FORMER	32239 (50.2%)	12228 (49.7%)	44467 (50.1%)
NEVER	15184 (23.7%)	6229 (25.3%)	21413 (24.1%)
Missing	2 (0.0%)	0 (0%)	2 (0.0%)
Systolic blood pressure			
Mean (SD)	132.16 (18.97)	131.77 (18.33)	132.05 (18.80)
Median [Min, Max]	131.00 [80.00, 260.00]	130.00 [80.00, 235.00]	131.00 [80.00, 260.00]
Missing	496 (0.8%)	202 (0.8%)	698 (0.8%)
Diastolic blood pressure			
Mean (SD)	75.83 (10.40)	75.98 (10.17)	75.87 (10.33)
Median [Min, Max]	76.00 [50.00, 162.00]	76.00 [50.00, 152.00]	76.00 [50.00, 162.00]
Missing	496 (0.8%)	202 (0.8%)	698 (0.8%)

Suppl Fig 1. Distribution of baseline LDL-C in 167505 veterans in VALOR-QI program

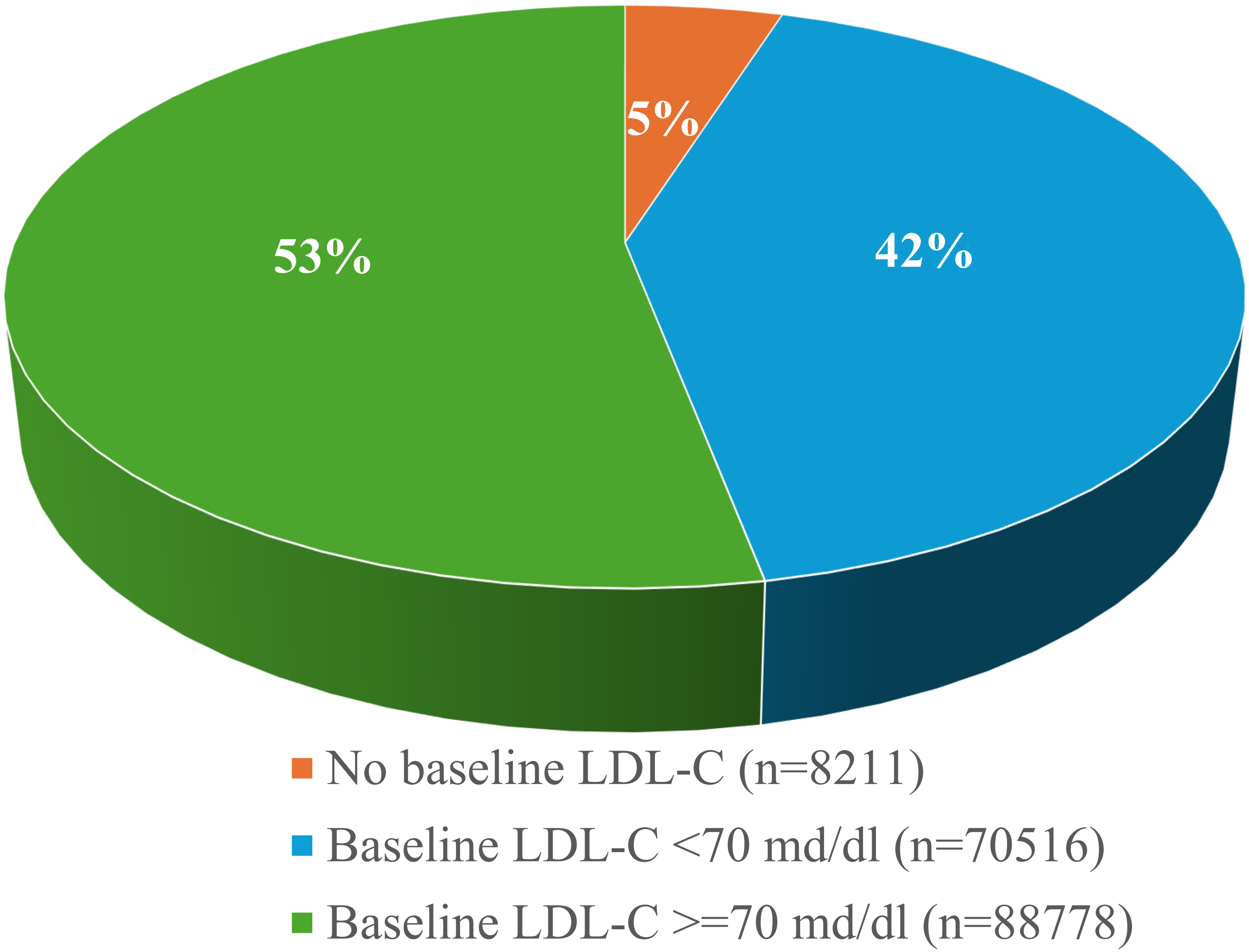

Suppl Fig 2. Change in LLT use between baseline and 24 months

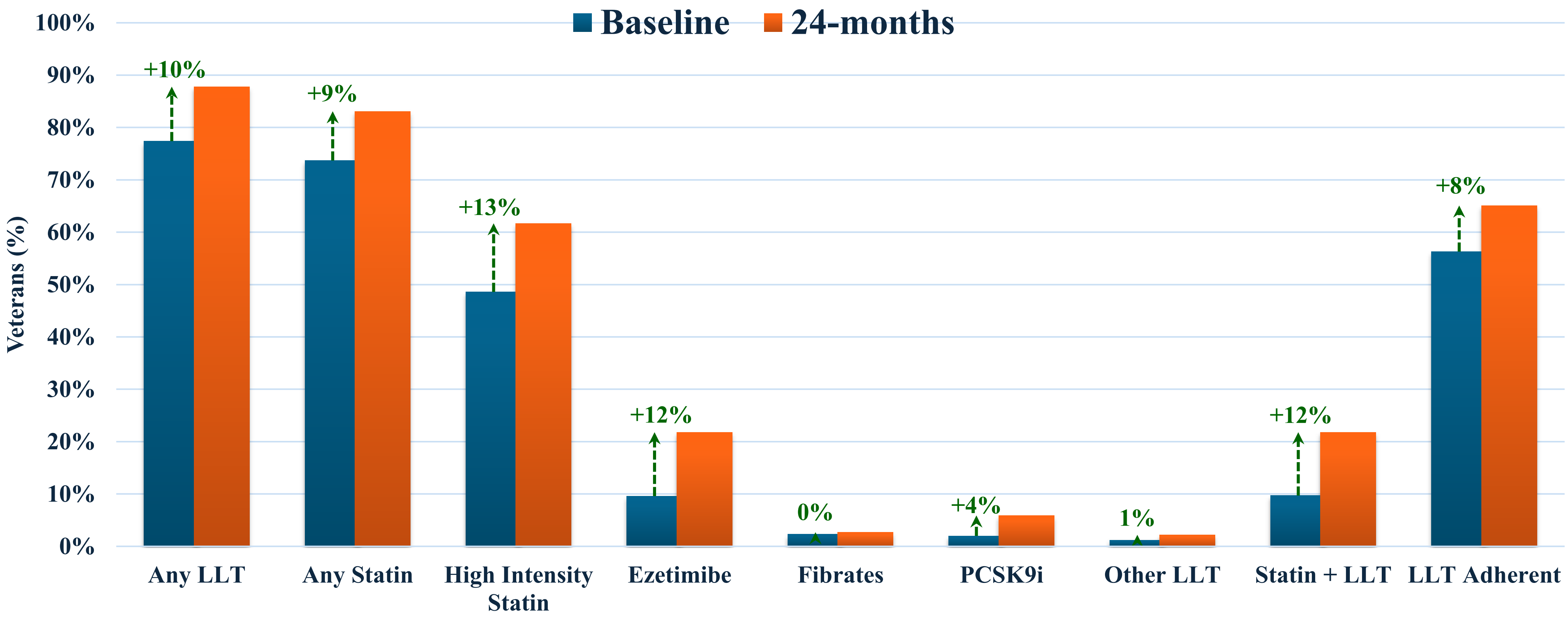

Type of lipid lowering therapy (LLT)

Suppl Fig 3. Mean change in LDL-C at 24 months by baseline LDL-C

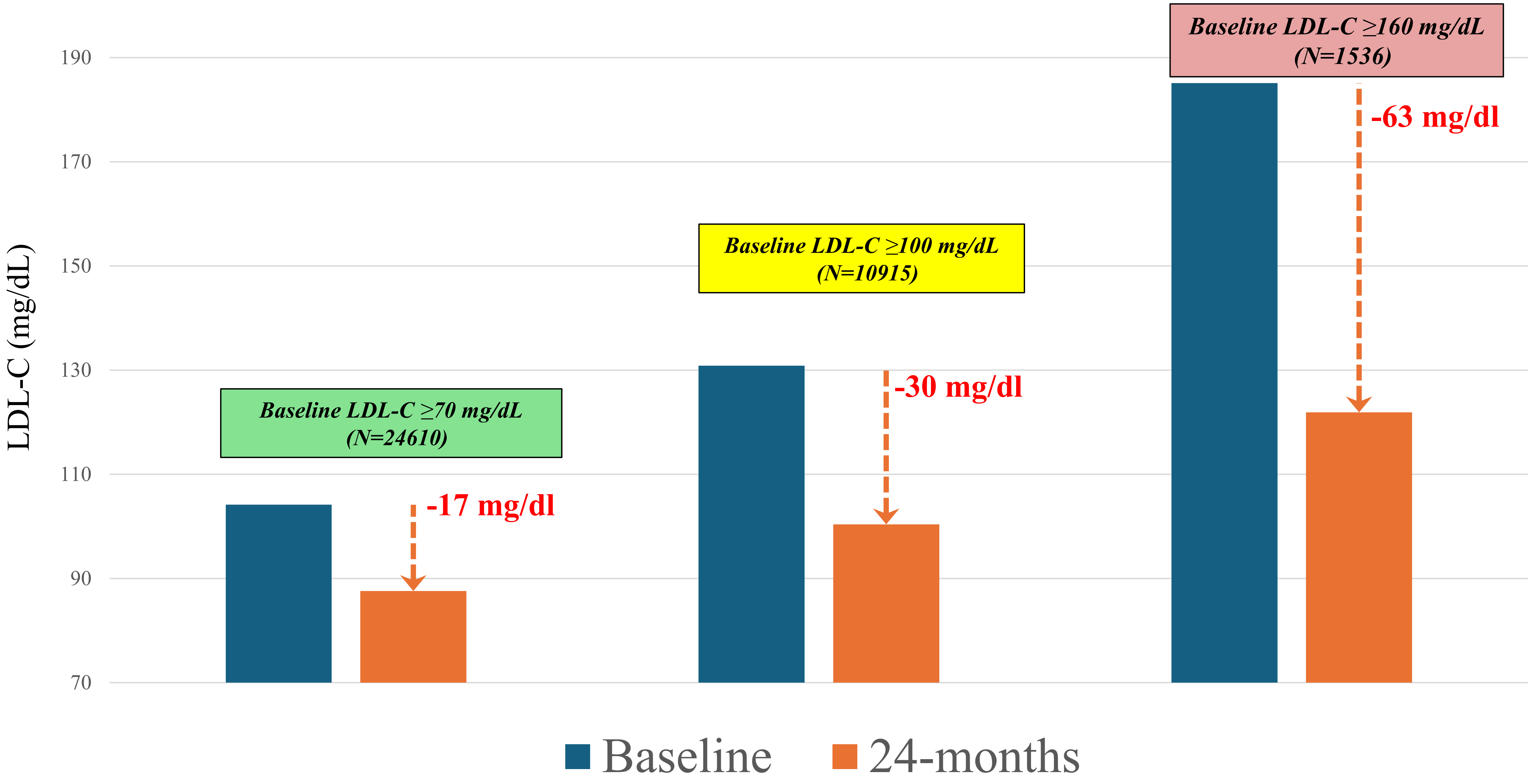

Research and reporting methodology

Revised **Standards for QUality Improvement Reporting Excellence** (**SQUIRE 2.0**) publication guidelines

Notes to authors

- The SQUIRE guidelines provide a framework for reporting new knowledge about how to improve healthcare.
- The SQUIRE guidelines are intended for reports that describe system level work to improve the quality, safety and value of healthcare, and used methods to establish that observed outcomes were due to the intervention(s).
- A range of approaches exists for improving healthcare. SQUIRE may be adapted for reporting any of these.
- Authors should consider every SQUIRE item, but it may be inappropriate or unnecessary to include every SQUIRE element in a particular manuscript.
- The SQUIRE glossary contains definitions of many of the key words in SQUIRE.
- The explanation and elaboration document provides specific examples of well-written SQUIRE items and an in-depth explanation of each item.
- Please cite SQUIRE when it is used to write a manuscript.

Text section and item name	Page/line no(s). info is located
**Title and abstract**	
1. **Title**	
Indicate that the manuscript concerns an initiative to improve healthcare (broadly defined to include the quality, safety, effectiveness, patient-centredness, timeliness, cost, efficiency and equity of healthcare).	1
2. **Abstract**	
a. Provide adequate information to aid in searching and indexing.	3
b. Summarise all key information from various sections of the text using the abstract format of the intended publication or a structured summary such as: background, local problem, methods, interventions, results, conclusions.	3
**Introduction: Why did you start?**	
3. **Problem description** - Nature and significance of the local problem.	5
4. **Available knowledge** - Summary of what is currently known about the problem, including relevant previous studies.	5
5. **Rationale** - Informal or formal frameworks, models, concepts and/or theories used to explain the problem, any reasons or assumptions that were used to develop the intervention(s) and reasons why the intervention(s) was expected to work	5-6

6. **Specific aims** - Purpose of the project and of this report.	6
**Methods: What did you do?**	
7. **Context** - Contextual elements considered important at the outset of introducing the intervention(s).	5-6
8. **Intervention(s)**	
a. Description of the intervention(s) in sufficient detail that others could reproduce it.	7
b. Specifics of the team involved in the work.	7-8
9. **Study of the intervention(s)**	
a. Approach chosen for assessing the impact of the intervention(s).	8-9
b. Approach used to establish whether the observed outcomes were due to the intervention(s).	8-9
10. **Measures**	
a. Measures chosen for studying processes and outcomes of the intervention(s), including rationale for choosing them, their operational definitions and their validity and reliability.	7-8
b. Description of the approach to the ongoing assessment of contextual elements that contributed to the success, failure, efficiency and cost.	7-8
c. Methods employed for assessing completeness and accuracy of data.	7

11. **Analysis**	
a. Qualitative and quantitative methods used to draw inferences from the data.	8-9
b. Methods for understanding variation within the data, including the effects of time as a variable.	8-9
12. **Ethical considerations** - Ethical aspects of implementing and studying the intervention(s) and how they were addressed, including, but not limited to, formal ethics review and potential conflict(s) of interest.	7
**Results: What did you find?**	
13. **Results**	
a. Initial steps of the intervention(s) and their evolution over time (eg, time-line diagram, flow chart or table), including modifications made to the intervention during the project.	9
b. Details of the process measures and outcomes.	9
c. Contextual elements that interacted with the intervention(s).	9
d. Observed associations between outcomes, interventions and relevant contextual elements.	9-10
e. Unintended consequences such as unexpected benefits, problems, failures or costs associated with the intervention(s).	10
f. Details about missing data.	9
**Discussion: What does it mean?**	
14. **Summary**	
a. Key findings, including relevance to the rationale and specific aims.	11
b. Particular strengths of the project.	12

15. **Interpretation**	
a. Nature of the association between the intervention(s) and the outcomes.	14
b. Comparison of results with findings from other publications.	11-12
c. Impact of the project on people and systems.	11-12
d. Reasons for any differences between observed and anticipated outcomes, including the influence of context.	13
e. Costs and strategic trade-offs, including opportunity costs.	-
16. **Limitations**	
a. Limits to the generalisability of the work.	12-13
b. Factors that might have limited internal validity such as confounding, bias or imprecision in the design, methods, measurement or analysis.	13
c. Efforts made to minimise and adjust for limitations.	9, 13
**Conclusions**	
a. Usefulness of the work.	14
b. Sustainability.	14
c. Potential for spread to other contexts.	14
d. Implications for practice and for further study in the field.	14
e. Suggested next steps.	14
**Other information**	

18. **Funding** - Sources of funding that supported this work. Role, if any, of the funding organisation in the design, implementation, interpretation and reporting.	2

Ogrinc G, et al. BMJ Qual Saf 2015;0:1–7. doi:10.1136/bmjqs-2015-004411
Downloaded from http://qualitysafety.bmj.com/ on January 2, 2017
